# Early Auditory Event Related Potentials Distinguish Higher-Order From First-Order Aversive Conditioning

**DOI:** 10.3389/fnbeh.2022.751274

**Published:** 2022-02-11

**Authors:** Prateek Dhamija, Allison Wong, Asaf Gilboa

**Affiliations:** ^1^Department of Psychology, University of Toronto, Toronto, ON, Canada; ^2^Rotman Research Institute, Baycrest, Toronto, ON, Canada

**Keywords:** EEG, ventromedial prefrontal cortex (vmPFC), second-order conditioning, value, learning, model-based choice, hippocampus, Pavlovian (classical) conditioning

## Abstract

Stimuli in reality rarely co-occur with primary reward or punishment to allow direct associative learning of value. Instead, value is thought to be inferred through complex higher-order associations. Rodent research has demonstrated that the formation and maintenance of first-order and higher-order associations are supported by distinct neural substrates. In this study, we explored whether this pattern of findings held true for humans. Participants underwent first-order and subsequent higher-order conditioning using an aversive burst of white noise or neutral tone as the unconditioned stimuli. Four distinct tones, initially neutral, served as first-order and higher-order conditioned stimuli. Autonomic and neural responses were indexed by pupillometry and evoked response potentials (ERPs) respectively. Conditioned aversive values of first-order and higher-order stimuli led to increased autonomic responses, as indexed by pupil dilation. Distinct temporo-spatial auditory evoked response potentials were elicited by first-order and high-order conditioned stimuli. Conditioned first-order responses peaked around 260 ms and source estimation suggested a primary medial prefrontal and amygdala source. Conversely, conditioned higher-order responses peaked around 120 ms with an estimated source in the medial temporal lobe. Interestingly, pupillometry responses to first-order conditioned stimuli were diminished after higher order training, possibly signifying concomitant incidental extinction, while responses to higher-order stimuli remained. This suggests that once formed, higher order associations are at least partially independent of first order conditioned representations. This experiment demonstrates that first-order and higher-order conditioned associations have distinct neural signatures, and like rodents, the medial temporal lobe may be specifically involved with higher-order conditioning.

## Introduction

Stimuli in the environment can acquire positive or negative value, if they appear in direct association with primary rewards or punishment (e.g., classical conditioning), however, this rarely occurs as an isolated process. Instead, it is thought that value is often inferred through complex higher-order associations. Higher-order associations form when intrinsically neutral stimuli that have acquired value through direct association with primary rewards or punishments, thus known as conditioned stimuli, are then associated with novel stimuli (Pavlov, 1927; Gewirtz and Davis, 2000). Through this process, higher-order associations enable the representation of important environmental stimuli and their inferred value, promoting flexible and adaptive behavior.

The formation of first-order associations between neutral and inherently valued stimuli is a pre-requisite for inference of value in higher-order relationships. In first-order conditioning, an unconditioned stimulus (US) with an intrinsic value elicits a natural behavior, unconditioned response (UR). The US is preceded by an initially neutral stimulus, known as the conditioned stimulus (CS). The CS is thought to acquire the value of the US (Pavlov, 1927; Gewirtz and Davis, 2000) and motivates behavior even in the absence of the US, which is known as the conditioned response (CR). In higher-order conditioning, this process is extended by one step as the CS with acquired value are associated with novel stimuli without value. These novel stimuli acquiring value are known as higher-order stimuli (HO) and can elicit a CR despite never being directly associated with the US (Pavlov, 1927).

While the formation of higher-order associations depends on the strength of their corresponding first-order associations, once robust higher-order associations are formed, higher-order associations are not simply an extension of first-order learning. Neural structures that represent reward value and motivation cannot independently support representations of intrinsic higher order relations (Wimmer and Shohamy, 2012; Gilboa et al., 2014; Gilboa and Moscovitch, 2021). Lesion studies suggest that first-order and higher-order associations are supported by partially overlapping, but distinct, neuroanatomical structures that seem to differ in their contributions to first-order and higher-order associations. The basolateral amygdala (BLA) and hippocampus are two such structures. Holland (2016) demonstrated that if both forward conditioned first-order training (CS → US) and forward conditioned (HO → CS) higher-order training occurred after lesion to the BLA, higher-order learning was impaired. However, if first-order training occurred with an intact BLA and only higher-order training occurred after lesion, then enhanced higher-order associations were formed (Holland, 2016). This suggests that the BLA is critical for first-order conditioning, but that once first-order conditioning is formed, higher-order associations can develop independently of the BLA. Furthermore, the absence of the BLA may enhance higher-order learning because it slows the extinction of first-order CSs during higher-order training (Lindgren et al., 2003; Holland, 2016). Conversely, in a study by Gilboa et al. (2014) hippocampal lesions did not affect forward auditory first-order conditioning (CS → US) but did severely impair both acquisition and retention of backward serial unimodal auditory higher-order conditioning (CS → HO). In addition to these neuroanatomical dissociations revealed by lesion studies, higher-order associations can become functionally independent of their corresponding first-order association, as demonstrated by persisting higher-order associations after extinction of the underlying first-order associations (Pavlov, 1927; Rizley and Rescorla, 1972; Rashotte et al., 1977; Rescorla, 1979, 1982; Cole et al., 1995). These studies utilize either appetitive or aversive USs with a a cross-modal presentation of CSs and HOs, with most studies utilizing a forward conditioning paradigm (CS → US; HO → CS), though some have used a backward conditioning paradigm (CS → US; CS → HO; Cole et al., 1995).

The above-described work suggests that first-order and higher-order can be dissociated by both behavioral responses and the neuroanatomical structures in rodents, consistent with a neural-psychological correspondence view of memory (NPRC; Gilboa and Moscovitch, 2021; cf. Hebscher et al., 2019). However, relatively few studies have investigated higher-order conditioning in humans (Pauli et al., 2019; Prével et al., 2019; Luettgau et al., 2021; see Honey and Dwyer, 2021; Lee, 2021 for a review). Even fewer have examined the neural processes of first-order and higher-order learning in humans. Using functional resonance magnetic imaging (fMRI) to examine sequential learning paradigms (CS1→ CS2 → US), the striatum and orbitofrontal structures have been implicated in learning distally predictive sitmuli (Pauli et al., 2019). These paradigms, however, present both the first-order and higher-order stimuli within the same training trials as the US, tapping gradual learning of complex temporal relationships among conditioned and unconditioned stimuli rather than transfer of value that had been acquired previously by the CS, in the absence of a US. Moreover, fMRI provides excellent spatial resolution but lacks high temporal resolution to examine differences between short timescale temporal features of first-order and higher-order learning. Electroencephalogram (EEG) provides such temporal resolution and has been used previously to examine higher-order conditioning in smokers using pre-established first-order visual stimuli as appetitive CS (e.g., cigarette packs) and simple geometric figures as higher-order stimuli (Littel and Franken, 2012). Higher order visual-visual associations in smokers led to increased evoked response potentials (ERPs) as early as 200–280 ms. over fronto-central electrodes whereas smoking-related first order conditioned stimuli produced a larger P3 component similarly distributed but starting later, from 300 ms. The earlier components of the ERP in which significant differences were elicited by higher-order conditioned stimuli is surprising. However, comparison of well-established, addictive first-order associations to novel higher-order associations may differ from higher-order learning that occurs soon after first-order learning has been established. Moreover, naturalistic smoking related cues are more visually complex than the simple figures used as CS2 by Littel and Franken (2012), which may partially account for the temporal difference.

In the current study, we aimed to determine if aversive auditory higher-order conditioning could be established in humans, by adapting our rodent paradigm where we demonstrated that backwards higher-order associations could be dissociated from first-order associations (Gilboa et al., 2014). Furthermore, we explored the accompanying electrophysiological activity to determine if the neural responses to first-order and higher-order associations were dissociable in the temporal and spatial domains. Participants were conditioned using consecutive (non-overlapping) auditory stimuli. First-order associations were formed between a neutral tone (CS+) and an aversive burst of white noise (US+; addition of aversive value is indicated by + and neutral value is indicated by –). Implicit anticipation of aversive stimuli (intrinsic or acquired) was measured by pupillary dilation (Korn et al., 2017). To compare these conditioned responses to a control, participants associated two neutral tones as the neutral conditioned stimulus (CS–) and neutral unconditioned stimulus (US–). Following the establishment of first-order conditioned associations, we paired the CS tones that had acquired value with distinct novel auditory stimuli (HO+/HO–) to form higher-order associations.

## Method

### Participants

A total of 16 healthy middle-aged adults were recruited for the study. Of these, one participant withdrew from the study and one participant’s data were lost due to technical error, leaving data from 14 individuals (9 males, 5 females, average age = 54.71 years, average education = 16.54 years). Participants were recruited using Baycrest Hospital research participant database, had no history of substance abuse, neurological or significant psychiatric disorders, had normal hearing and normal or corrected to normal vision and were between 40 and 65 years old. Participants also completed a questionnaire that included health questions and questions on age, gender, and education level. This study was approved by the Research Ethics Board at the Rotman Research Institute/Baycrest Hospital. All participants provided written and informed consent before the experiment and were monetarily compensated at rate of $15 per hour plus travel expenses.

### Stimuli and Stimulus Presentation

We generated seven distinct auditory stimuli by varying frequency (350, 500, 750, and 1000 hz) and waveform (sawtooth and sine) to establish within sensory modality conditioning effects. These were similar to stimuli we have used in our previous rodent studies examining higher-order conditioning (Gilboa et al., 2014, 2019). In addition, a 500 ms burst of 100 dB white noise was used as the aversive unconditioned stimulus (US+). The peak amplitude of the US+ was 40 dB higher than that of the conditioned tones to ensure that it was sufficiently aversive. The stimuli were randomly assigned to each condition^1^.

To ensure that stimuli were not initially different, we compared naïve ratings of the stimuli. As expected, the US+ stimulus was rated as significantly more aversive than US–, whereas no other stimuli (CS+ compared to CS– or HO+ compared to HO–) were rated significantly different (see Supplementary Analysis 1 and Supplementary Figure 1).

Furthermore, after data collection, we compared participants’ pupil responses in the first seven presentations of the stimuli to test whether there were pre-learning differences in responses to the physical characteristics of the auditory tones. We observed significantly larger pupil dilation in response to US+ compared with US– (Supplementary Figure 2), consistent with the aversive nature of these stimuli, but no significantly larger pupil responses for CS+ compared with CS– (Supplementary Figure 3) or for HO+ compared with HO– (Supplementary Figure 4). This suggests that unlike US+, larger pupil dilation for CS+ and HO+ found later in the experiment are likely acquired through training rather than inherent to the physical characteristics of the stimuli.

The experiment and cover task were deployed using E-prime 1.2 (Psychology Software Tools, PA, United States). Visual cues appeared on an LCD monitor with a refresh rate of 60 hz. E-prime delivered meta-trial information to the EEG and eye tracker when initiating each trial. The experiment was conducted in a sound isolated room. Auditory stimuli were delivered using ER-3A insert earbuds (Etymotic Research, Elk Grove, IL, United States). Acoustic tubing was used to avoid electromagnetic artifacts caused by stimulus delivery, similar to previous auditory EEG experiments (Aiken and Picton, 2008; Campbell et al., 2012). Participants were seated in a comfortable chair with cushions for support.

### Experimental Design

The current study proceeded over 2 days in five phases with participants completing phase one (first-order conditioning) on the first day and the remaining four phases (first-order reminder, higher-order conditioning, first-order testing, and higher-order testing) on the second day.

Participants completed a tone rating task at the start and end of phase one, phase two, and phase three to examine the prior and post rating of stimuli. We examined this information for three reasons: to test reactivity to the US, to test if conditioned stimuli were inherently aversive, and to test if there were shifts in explicit ratings of stimuli’s aversiveness after each conditioning phase. Given the non-declarative nature of Pavlovian conditioning (Squire and Zola, 1996) we did not necessarily expect to observe changes in ratings that would correspond with autonomic reactivity changes, and in fact used a perceptual cover task to maximize attention to the stimuli and their relationships but minimize intentional encoding of the conditioned associations.

In phase one, first-order conditioning, participants were conditioned while performing a cover task to form first-order associative relationships, pairing CS+ with US+ and CS– with US–. This cover task was used for phases one through three. In phase 1 (1st day) participants responded to the cover task using the left and right buttons on the mouse to indicate if the tones originated from the same direction (left) or different directions (right). In phase 2 (2nd day), first-order reminders, participants repeated a shorter version of phase one as a reminder of the first-order CS± and US± pairs. In phase 3, higher-order conditioning, participants established higher-order conditioning relationship between CS± and the HO±. This was similar to phase one except that the US± tones were replaced with novel neutral tones intended to become the HO±. In phase four, we tested participants’ reactivity to the CS± tones and in phase five, we examined participants’ reactivity to the HO± tones.

We measured participants’ pupil dilation to evaluate their evoked physiological responses throughout all phases of the study. Pupillometry has been shown to be an effective measure of Pavlovian conditioned responses in humans (Reinhard and Lachnit, 2002) and has been suggested to be one of the best methods to discriminate CS+/CS– conditioned responses (Ojala and Bach, 2020; see Finke et al., 2021 for review and meta-analysis). To measure evoked neural responses, continuous EEG was recorded throughout the experiment (more detail below).

#### Cover Task and Trial Overview

Participants were informed that the task was perceptual in nature, and their goal was to determine the directional origin of the stimuli. They completed this cover task throughout the study to avoid intentional learning of the associations, and at the same time to ensure they remain engaged with the stimuli and, crucially, the relationships between them. On each trial, participants viewed a fixation cross for a random duration between 3000 and 6000 ms (across all phases, *M* = 4718.04 ms; *SD* = 862.29 ms) before the onset of the two consecutive auditory stimuli to collect a stable baseline. Participants continued to fixate on the cross while the auditory stimuli were presented. In the conditioning and reminder phases (phases 1, 2, and 3), two consecutive, no gap, non-overlapping stimuli were presented. Whereas for testing phases (phases 4 and 5), a single stimulus was presented. At the end of each trial, participants were asked to indicate if the two tones originated from congruent or incongruent directions, or, in the testing phases, if the single tone had originated from the left or right. After both stimuli had been presented, a decision screen appeared prompting participants to indicate if the stimuli originated from congruent or incongruent directions with a mouse. A reminder of the left/right response mapping appeared and remained on screen until they made their decision. Participants had unlimited time to make their decision.

#### Day One

##### Tone Rating Task

Participants performed a tone-rating task before and after phase 1, 2, and 3. Participants rated four 3-s CS± and US± tones on unpleasantness on a Likert-like scale from 1 to 9 (1 = Neutral, 9 = Extremely Unpleasant). In Phase 1 and Phase 2, tones were presented in the following order: CS–, CS+, US–, US+. In phase 3, tones were presented in the following order: CS–, CS+, HO+, US–.

##### Phase One: First-Order Conditioning

Following the tone-rating task, participants incidentally learned first-order associative relationships between the CS± and US±. To ensure that participants continued to pay attention to the task while being unaware of the conditioning procedure, they were given the cover task described above. On each trial, participants viewed a fixation cross, heard two consecutive auditory stimuli while still fixating, followed by a decision screen for the tone direction cover task until they indicated their choice by key press. Participants were presented with 80 trials (32 CS+: US+, 32 CS–: US–; 16CS+: CS+), in randomized order for each participant. The duration of the CS± varied between 3500, 4500, 5500, or 6500 ms and was counterbalanced to ensure that each length and congruency of the stimuli were presented equally for CS+: US+ and CS–: US– trials. On 16 of the trials, CS+ stimuli were presented twice consecutively instead of the CS+ being followed by presentation of the US+. This partial reinforcement schedule resulted in reduced predictability of the CS±: US± associations which had three benefits: reduction of explicit learning of the associations, prevention of habituation to the US+ and enhanced acquisition of first-order associations. Partial reinforcement schedules have been suggested to produce more robust higher-order conditioning (Gewirtz and Davis, 1997, 2000; Kamil, 1969). The duration of the US± stimuli were fixed at 500 ms.

#### Day Two: Phase Two to Five

##### Phase Two: First-Order Conditioning Reminder

Participants were presented with a total of 20 trials from phase one to reactivate memories of the first-order conditioning pairs from the previous day. This consisted of 8 CS+: US+ trials, 8 CS–: US– trials and 4 CS+: CS+ trials.

##### Phase Three: Higher-Order Conditioning

Following the tone-rating task, the higher-order relationships between the CS± and HO± were presented to the participants. On each trial, participants viewed a fixation cross, heard the CS and HO pair and followed by a decision screen, indicating whether the pair of tones originated from congruent or incongruent directions. Participants were presented with 48 trials (24 CS+: HO+ trials and 24 CS–: HO– trials) in randomized order for each participant (intertrial interval *M* = 4767.63 ms, *SD* = 861.45 ms). To prevent expectancy and maintain stimulus salience, the duration of the CS± were varied between 3000 and 6000 ms. The duration of the HO± was fixed at 4000 ms.

##### Phase Four: First-Order Stimuli Testing

Participants completed 50 trials presented in random order to examine their response to the CS± stimuli (24 CS+, 24 CS–, 2 CS+: US+). On each trial, participants heard either the CS+ or CS– for 4000 ms (intertrial interval *M* = 4698.93, *SD* = 885.27 ms). A similar cover task to the one used in phases one to three was given to participants. Participants were asked to indicate the origin of the tone (left or right) using left or right mouse clicks. On two of the 50 trials, participants were presented with first-order reminder trials; the US+ (100 db burst of white noise) was presented immediately after the CS+. These reminder trials were employed because training on high-order conditioning is known to lead to extinction of the first-order associations (Gewirtz and Davis, 2000) and we expected reminder trials to mitigate behavioral extinction of CS+ responding. The reminder trials were excluded from pupillometry and EEG analyses.

##### Phase Five: Higher-Order Stimuli Testing

Similar to phase four, participants completed 48 trials (24 HO+; 24 HO–) presented in random order to examine their response to the HO± stimuli (intertrial interval *M* = 4728.15, *SD* = 865.65). On each trial, participants heard either the HO+ or HO– for 4000 ms. A similar cover task used as the one used in phase four was given to participants where they were asked to indicate the origin of the tone (left or right) using the mouse.

### Data Analysis

There was one case where a participant’s EEG data were corrupted and were therefore not included in the analysis of phase 3. The participant’s data were included for analysis in the other phases.

There were three cases where a participant’s pupillometry was corrupted in phase 1 and one case in phase 2. Those participant’s data were removed from analysis of the affected phase.

#### Pupillometry Apparatus and Analysis

Measurements of the size of participants’ left pupil were acquired using Eyelink 1000 (SR Research; Ottawa, ON, Canada) with a sampling rate of 500 hz. Prior to each phase of the experiment, calibration and drift correction were performed. Cohen and Hershman Analysis Pupil (CHAP version 1.5), a MATLAB (ver. R2020a) open-source software, was used for pre-processing and analysis of pupillometric data (Hershman et al., 2019).

Preprocessing of pupil data using CHAP included four steps to ensure that data were viable for analysis. The first was the exclusion of outlier samples with *Z*-scores exceeding ± 3. *Z*-scores were calculated for each trial using the mean and standard deviation of the 1500 ms baseline period prior to stimuli presentation. Second, outlier trials were excluded if >25% samples were missing. We excluded 14.18% of trials in this way. Third, blinks were detected by an algorithm which identifies sharp decreases and increases that precedes and follows a missing pupil during blinking (Hershman et al., 2018). Missing data that were caused by blinks were corrected using linear interpolation. The fourth pre-processing step was the exclusion of participants who were missing 50% or more of trials in either condition. Based on these four preprocessing steps, one participant was excluded from phase 3, two participants were excluded in phase 2, and three participants were excluded from phase 5. This resulted in the following participants included in the analysis for pupillometry: 11 participants in phases 1 and 5, 12 participants in phases 3 and 4, and 13 participants in phase 2. Prior to analysis, trials were aligned using the onset of the first stimulus for each trial and converted to change scores based on each trials baseline (1500 ms pre-stimulus onset). Each trial’s data was converted to a *z*-score by using the 1500 ms pre-stimulus onset period as the expected mean and standard deviation.

Analysis was conducted by comparing the relative *z*-scored pupil size change between the two conditions during the post-stimulus onset period of interest (220 ms post-stimulus onset to the end of the trial; Hershman et al., 2018). For each phase, a series of Bayesian paired sample *t*-tests were conducted over the post-stimulus period of interest. This meant that each sample, taken every 2 ms, was compared between the two conditions using a Bayesian paired-samples *t*-test. We used a default Cauchy prior width of *r* = 0.707 for effect size on the alternative hypothesis over the null hypothesis (Rouder et al., 2012). The Bayes Factor (*BF*) threshold was 3, a value that represents substantial evidence (Jeffreys, 1961). In this case, this means that the measured pupil sizes from two conditions are not the same. This type of analysis has been used in recent studies comparing pupil dilation (Papesh and Pinto, 2019; Hershman et al., 2021).

#### Evoked Response Potentials Recordings and Analysis

Continuous EEG was recorded using the Biosemi Active Two acquisition system (BioSemi V.O.F., Amsterdam, Netherlands) and a montage of 72 electrodes, with a Common Mode Sense (CMS) active electrode and Driven Right Leg (DRL) passive electrode serving as ground. In addition to the 64-channel scalp electrode cap based on the 10/20 system, we used eight facial electrodes placed below the hairline (both mastoid points, both pre-auricular points, outer canthus of each eye, and inferior orbit of each eye) to measure ocular movement and ensure even coverage of the whole scalp. Equal scalp coverage ensured that we were able to use an average of all scalp EEG channels as a reference for each channel for ERP analysis. Neural activity was digitized continuously at a rate of 512 Hz with a bandpass of 0.16 hz–100 Hz and stored for offline analysis. Brain Electrical Source Analysis software (BESA, version 6.1; MEGIS GmbH, Gräfelfing, Germany) was used to perform analysis.

The continuous EEG were visually inspected for channels displaying faulty recordings and these were either interpolated or ignored (if they were around the rim of the cap) and large muscle artifacts were tagged. An independent component analysis (ICA) was then performed on a 40 s time window to parse any spatial topographies of artifact-related patterns of activity (e.g., horizontal or vertical eye movements, eyeblinks, EKG activity, etc.). These were identified and subtracted from the continuous EEG. A 0.53 high bandpass digital filter (forward, 6 dB/octave) was applied. The continuous EEG files were segmented into 900 ms epochs (including a 100 ms pre-stimulus window and the first 800 ms. of the stimulus presentation) and re-referenced to a common average reference. Trials were sorted by phase of experiment and stimulus type: phase 3 first-order tones (CS+, CS–), phase 4 first-order tones (CS+ or CS–) and phase 5 higher-order testing (HO+ or HO–). Note that ERPs corresponded to the initial time window of each trial and so evoked responses during conditioning were always to the first stimulus in each pair, before the HO appeared in phase 3. ERPs were digitally low-pass filtered to attenuate frequencies of >20 Hz and averaged for each condition. ERP amplitudes were measured relative to the mean amplitude over the pre-stimulus interval. Statistical analyses of ERP waveform differences were performed for 0–800 ms. using BESA Statistics 2.0, which includes a spatio-temporal permutation-based correction for multiple comparisons. We used a cluster alpha of 0.05, 1000 permutations, with clusters defined using the default channel distance of 4 cm.

An iterative 3D source imaging method, CLARA (Classical Low-Resolution Electromagnetic Tomography Analysis Recursively Applied), was used for source estimation of surface-level evoked response components that showed significant difference in amplitude. The CLARA approach applies the LORETA algorithm iteratively localizes activity to the constrained regions identified from the previous solution. Three iterations were computed using the default voxel dimension of 7 mm^3^ and 1% regularization constant. The solution was computed using an adult realistic head model in BESA 6.1 and registered against the standardized BESA finite element model, which was created from the average of 24 individual anatomical magnetic resonance imaging (MRIs) in the Talairach-Tournoux coordinate space. Condition differences in source solution of the evoked responses were tested using a parameter-free permutation paired *t*-test in combination with data clustering to correct for multiple comparisons of the averaged source across the time window of significant surface-level component difference, implemented in BESA statistics 2.0.

#### Correlation of Pupil Size and Neural Responses

To examine if greater pupil responses were associated with a more extreme electrophysiological response, the *z*-score of mean pupil size from segments that were found to be significantly different were extracted and correlated with individual cluster scores from the ERP scalp analysis for each participant using Jamovi, a GUI for R (R Core Team, 2014; The Jamovi Project, 2020).

## Results

Below we first describe participants’ pre-task subjective ratings of each stimulus. We then describe the autonomic pupil and neural responses to first-order stimuli followed by the autonomic pupil and neural responses to higher-order stimuli.

### Cover Task and Subjective Tone Rating of Stimuli

Participants’ responses to the cover task suggested that participants remained engaged with the task as they had responded on every trial for all tasks. Furthermore, participants’ response accuracy during conditioning phases (phase 1, phase 2, and phase 3) suggest that they were attending to the relation between the two stimuli and that the task was sufficiently challenging so as to avoid ceiling effects, and not too challenging so as to avoid floor effects (proportion correct, *M* = 0.72, *SD* = 0.42).

To ascertain whether the US+ tone was intrinsically more aversive than other stimuli, we compared participants’ ratings of each tone prior to the start of the experiment. A one-way repeated measures ANOVA revealed a main effect of tone type, *Greehouse–Geisser F*(5,50) = 10.10, *p* < 0.001, η^2^ = 0.503. Follow up tests revealed that participants rated the US+ tone as significantly more aversive than all other tones, *p* < 0.001. All other tones were not rated as significantly different than each other, all *p’s* > 0.05. Thus, participants found the US+ more aversive than the other stimuli prior to any conditioning, and the other tones were not inherently different subjectively.

In addition, comparison of subjective ratings of tones before or after the task revealed no significant differences (all *p’s* > 0.05). Collapsing pre-and post- tone ratings, participants rated the CS– tone as more aversive than the HO– tone in phase 3, though stimuli were never rated significantly different than their complementary tone in valence (i.e., no difference between CS+ and CS– and HO+ and HO–; see Supplementary Analysis 1 and Supplementary Figure 1 for more detail).

### Behavioral Responses to First-Order Stimuli: Pupillometry

We predicted that participants would demonstrate greater pupil dilation for the stimuli associated with the acquired aversive value, i.e. greater pupil dilation in the CS+ and HO+ conditions than the CS– and HO– respectively.

#### Pupil Responses to First-Order Stimuli During Phase 1: First-Order Conditioning

**FIGURE 1:**
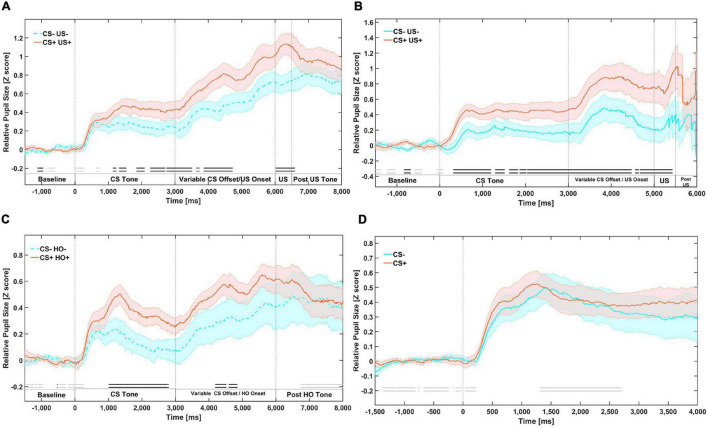
Mean relative pupil dilation (*z*-score) responses during first order tones in four different phases. **(A)** Phase 1: First-order conditioning on day 1. **(B)** Phase 2: First-order reminders on day 2. **(C)** Phase 3: Higher-order conditioning. First-order stimuli are paired with higher-order stimuli. **(D)** Phase 4: First-order testing. First-order stimuli presented alone. Meaningful differences (BF10 ≥ 3) are indicated by an opaque double line along the X axis of each panel of the figure, above the segment labels. Evidence in favor of the null hypothesis is indicated by faded double line along the X axis of each panel of the figure. The dark line in color represents the mean of that condition (orange for CS+/US+ trials and blue for CS–/US– trials). The lighter color bands surrounding the darker colored lines represent Standard Error of the Mean.

Our analysis suggests that there is substantial evidence (*BF_10_* ≥ 3) for meaningful differences in mean relative pupil dilation between the aversive and neutral tones from approximately 1100 to 1800 ms, and from 2000 to 3000 ms during the CS only presentations (see Figure 1A). Note that these differences reflect the gradual acquisition of value by the CS (see Supplementary Figure 3), but despite this there was evidence for conditioning. US onset was variable and occurred at the offset of the CS. There were differences from 3000 to 4750 ms during a time window where either the CS continued or a US may have appeared (mixed), and between approximately 6500 and 7000 ms during a time only a US was present (see Supplementary Figure 2 for onset aligned responses to US stimuli only early in the phase 1).

#### Pupil Responses to First-Order Stimuli During Phase 2: First-Order Reminders

Participants underwent a block of additional first-order conditioning trials after returning for the second day of the experiment. Our analysis suggests that there was substantial evidence (*BF_10_* ≥ 3) for meaningful differences in mean relative pupil dilation between the CS+ and CS–, which arose from approximately 330 ms and remained for over 5 s throughout the CS only and CS/US mixed time window until 5420 ms post onset of the CS, as well as throughout the US only time window (Figure 1B). This suggests robust maintenance of the value acquired the previous day, as this block was much shorter than phase 1.

#### Pupil Responses to First-Order Stimuli During Phase 3: Higher-Order Conditioning

**FIGURE 2:**
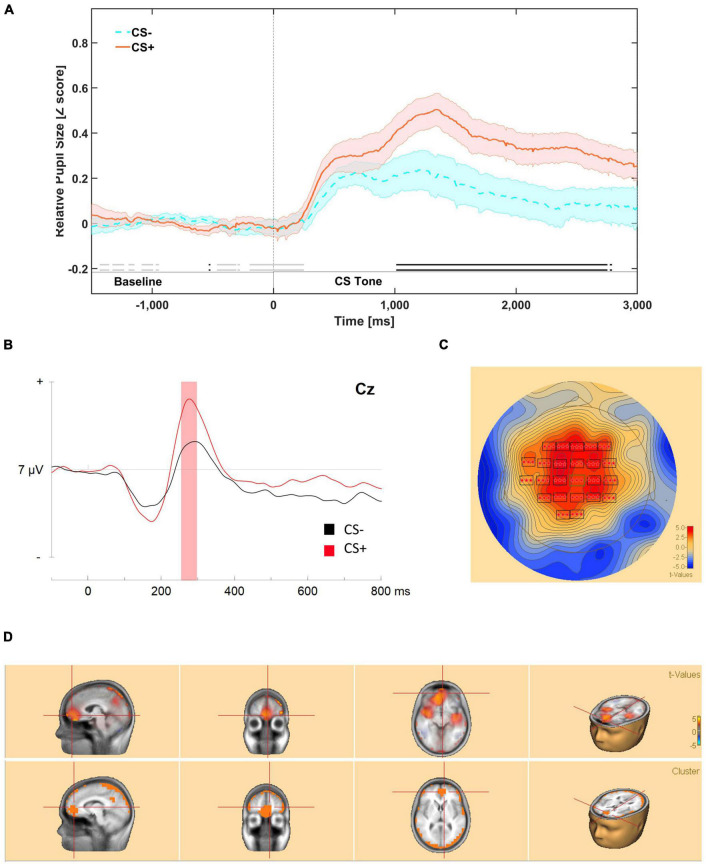
**(A)** Mean relative pupil dilation (*z*-score) responses during first order tones in phase 3, between –1500 ms (pre-stimulus onset) and 3000 ms (post-stimulus onset). Meaningful differences (BF_10_ ≥ 3) are indicated by an opaque double line along the X axis of the figure, above the segment labels. Evidence in favor of the null hypothesis is indicated by faded double line near the bottom of the figure. The dark line in color represents the mean of that condition (orange for CS+/US+ trials and blue for CS–/US– trials). The lighter color bands surrounding the darker colored lines represent Standard Error of the Mean. **(B)** ERP response to first-order stimuli presented during phase 3: higher-order conditioning at Cz. Bootstrap cluster analyses revealed a significant difference from 240 to 300 ms. **(C)** Mean potential distribution maps average across participants (*n* = 14 at scalp level. The significant positive modulation peaks bilaterally in frontal-parietal areas. **(D)** Source estimation analysis was conducted from 245 to 300 ms encompassing the significant greater modulation identified in source estimation. A positive significant source was identified right medial prefrontal cortex.

Our analysis suggests that there was substantial evidence (*BF*_10_ ≥ 3) for meaningful differences in mean relative pupil dilation from 1050 to 2700 ms during presentation of the CS (Figures 1C, 2A).

#### Pupil Responses to First-Order Stimuli During Phase 4: First-Order Testing

Conditioned pupil dilation to CS+ pupil dilation responses appear to have been extinguished as there are no longer meaningful differences (*BF*_10_ ≥ 3) in mean relative pupil dilation between the CS+ and CS– throughout the presentation of the CS+ (Figure 1D).

### Neural Responses to First-Order Stimuli Presented During Phase 3: Evoked Response Potentials

Evoked responses to auditory cues for both conditions revealed the typical well-established cortical auditory components described in the literature (Winkler et al., 2013; Remijn et al., 2014). These include a P1, N1 and an early P3 obligatory early response complex, which in this study peaked at roughly at 75, 125, and 275 ms, respectively (Winkler et al., 2013). The P1 is thought to reflect initial pre-attentive arousal to the auditory stimulus (Winkler et al., 2013), the N1 is thought to reflect pre-attentive representations of auditory stimulus features, including mnemonic characteristics (e.g., mismatch negativity; Molholm et al., 2005). Finally, the P3 is a marker of attentional capture (early) and of task-relevant stimulus identity processing (late; Winkler et al., 2013). As mentioned previously, we examined neural responses to first-order stimuli during presentation of first-order stimuli during phase 3: higher-order conditioning. The two reasons for this decision were: (1) first-order responses had extinguished by phase 4 (first-order testing) and (2) there were not adequate number of trials in phase 2 (first-order reminders), for fully powered bootstrap cluster analyses, however, see Supplementary Analysis 2 and Supplementary Figure 5 for phase 4 analyses.

#### Scalp Analysis

Permutation based analyses correcting for temporal and spatial extents of the ERP waveforms revealed a significant difference from 240 to 300 ms, with a greater positive peak in response to CS+ tones. This cluster encompassed electrodes bilaterally in frontal (F1, Fz, F2, F4, FC1, FCz, FC2, FC4), central (C3, C1, Cz, C2, C4), and parietal areas (CP3, CP1, CPz, CP2, CP4 P1, Pz). Within the temporal extension of the significant cluster, the peak response was centered on frontal, and central electrodes (Fz, FCz, Cz) throughout the response, cluster-based statistics, *p* < 0.001 (Figures 2B,C).

There was a significantly greater negative peak, peaking primarily – in the parietal areas (CP3, CP1, CPz, P3, Pz, P2, PO3) from 665 to 750 ms, cluster-based statistics, *p* = 0.038.

#### Source Analysis

Source estimation analysis was conducted on the ERP segment where significant greater positive modulation was identified in scalp analysis (240–300 ms). These cluster based bootstrap analyses revealed one significant positive cluster in the dorsal anterior cingulate area (BA 32) encroaching into the left anterior medial prefrontal cortex (BA 10), and onto other medial frontal areas including right amygdala cluster-based statistics, *p* < 0.0001 (Figure 2D).

### Correlations Between Pupil and Neural Responses to First-Order Stimuli During Phase 3

We conducted an exploratory correlation analysis of behavioral and neural responses to first-order tones presented in phase 3. There was a significant correlation between mean pupil dilation *z*-score during the maximal CS+/CS– difference (1050–2700 ms) and the significant cluster from the bootstrap analysis of evoked responses (240–300 ms) for CS+, *r*(12) = 0.643, *p* = 0.002, but not CS–, *r*(12) = 0.419, *p* = 0.175.

### Behavioral Responses to Higher-Order Stimuli During Phase 5: Pupillometry

We predicted that participants would demonstrate greater pupil dilation for the stimuli associated with the acquired aversive value. We predicted that participants would demonstrate greater pupil dilation in the HO+ conditions than the HO– respectively.

**FIGURE 3:**
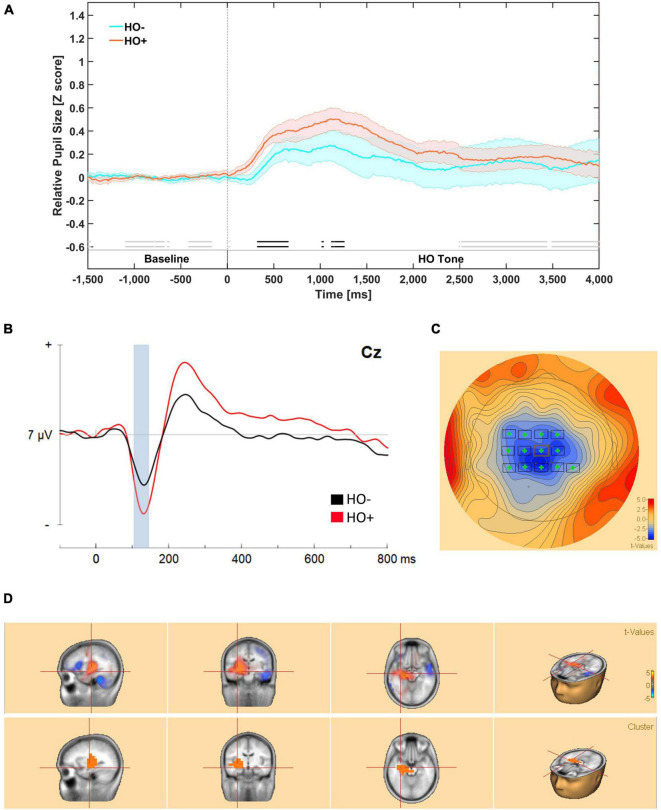
**(A)** Mean relative pupil dilation (*z*-score) responses during higher-order tone presentation in phase 5. Meaningful differences (BF_10_ ≥ 3) are indicated for by an opaque double line near the bottom of the figure above the segment labels. Evidence in favor of the null hypothesis is indicated by faded double line near the bottom of the figure. Meaningful differences are observed from 400 to 1500 ms post-stimulus onset. The dark line in color represents the mean of that condition (orange for CS+/US+ trials and blue for CS–/US– trials). The lighter color bands surrounding the darker colored lines represent Standard Error of the Mean. **(B)** Evoked responses to higher order tones revealed from bootstrap cluster analysis. Evoked responses waveform measured at Cz to higher order tones. Bootstrap cluster analyses revealed a significant difference from 100 to 155 ms. **(C)** Mean potential distribution map averaged across participants (*n* = 14) at scalp level. The significant negative modulation peaks bilaterally in central-parietal electrodes. **(D)** Source estimation from 100 to 135 ms revealed two significant clusters. A significant negative source encompassed the left parahippocampal area (BA 36) and the left Hippocampus (BA 54).

Our analysis suggests that there was substantial evidence (*BF_10_* ≥ 3) for meaningful differences in mean relative pupil dilation between the HO+ and HO– from approximately 320 to 650 ms and approximately 1300 to 1400 ms during the presentation of the tone (Figure 3A).

### Neural Responses to Higher-Order Stimuli During Phase 5: Evoked Response Potentials

#### Scalp Analysis

Evoked responses to auditory cues for both conditions matched the pattern of a P1, N1, and early P3 early response complex described in the literature (Woodman, 2010; Winkler et al., 2013) at roughly 75, 125, and 225 ms, respectively.

Permutation based analyses correcting for temporal and spatial extents of the ERP waveforms revealed a significant difference reflecting greater negative modulation in response to HO+ tones that encompasses N100, peaking predominantly in the left hemisphere in frontal area (FC3, FC1, FCz, FC2) central area (C3, C1, Cz, C2, C4, CP3, CP1, CPz, CP4) from 100 to 145 ms; cluster-based statistics *p* = 0.04 (Figures 3B,C).

#### Source Analyses of Evoked Response Potentials to Higher-Order Stimuli During Phase 5

Source estimation analysis was conducted from 100 to 145 ms encompassing the significant greater negative modulation identified in scalp analysis.

A significant source cluster reflecting greater positivity for HO+ compared to HO– encompassed the right hippocampus, while also encroaching onto the left Parahippocampal area (BA 36), *p* = 0.004 (Figure 3D).

### Correlations Between Pupil and Evoked Response Potentials Responses to Higher-Order Stimuli During Phase 5

**FIGURE 4:**
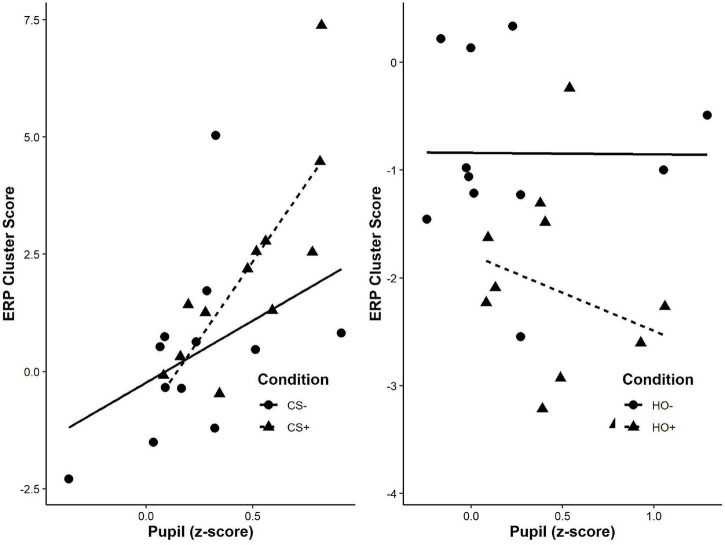
Correlation of mean pupil *z*-score and ERP cluster score for each participant comparing the valence of the effect (+: aversive represented by triangles; –: neutral represented by circles) for first-order (left) and higher-order stimuli (right). A significant positive correlation between mean pupil dilation z-score during the maximal CS+/CS− difference (1050–2700 ms) and the significant cluster from the bootstrap analysis of evoked responses (240–300 ms) for CS+, *r*(12) = 0.643, *p* = 0.002, but not CS−, *r*(12) = 0.419, *p* = 0.175. No such correlation was detected in response to higher-order stimuli.

We conducted an exploratory correlational analysis to determine if mean pupil dilation and significant clusters from ERPs were correlated. We did not find significant correlations between mean pupil dilations and evoked responses for either HO+, *r*(11) = 0.013, *p* = 0.696, or HO–, *r*(11) = –0.181, *p* = 0.594 (Figure 4).

### Correlation Between First-Order and Higher-Order Behavioral Responses

To determine if the strength of responses to higher-order stimuli were dependent on strength of responses to first-order stimuli. We conducted a correlation between mean pupil dilation responses for first-order stimuli during phase 3, and higher-order stimuli during phase 5. To control for potential different baseline pupil responses, we took a difference score between the stimuli associated with an aversive outcome and the stimuli associated with a neutral outcome (CS^difference^ = CS^+^ – CS^–^ and HO^difference^ = HO^+^ – HO^–^). We did not find significant correlations between mean CS^difference^ and HO^difference^, *r*(13) = 0.370, *p* = 0.213.

## Discussion

In the current study, we demonstrated that distinct behavioral and neural responses to higher-order and first-order stimuli could be identified in human participants. Participants acquired and retained first-order and higher-order associations, indexed by greater pupil dilation in response to CS+ and HO+ stimuli when compared to CS– and HO– stimuli, respectively. Evoked responses to first-order and higher-order stimuli shared the typical well established cortical auditory components including a P1, N1 and early P3, however, first-order and higher-order neural responses could be differentiated by the auditory component that was responsive to their acquired value. The later auditory component (early P3) uniquely discriminated acquired value for first-order stimuli (CS+ from CS–) whereas an earlier auditory component (N1) uniquely discriminated acquired value for higher-order stimuli (HO+ from HO–). First-order acquired aversive associations revealed greater positivity bilaterally over the central parietal scalp area during early P3, specifically from 240 to 300 ms. Conversely, higher-order acquired associations displayed an earlier greater negativity, the N1 component, specifically from 100 to 145 ms over the central scalp area. Source estimation models revealed distinct sources for these two components associated with first-order and higher-order conditioning. While the CS+ early P3 likely originated from left anterior mPFC, and amygdala, the HO+ N1 was estimated to arise from the parahippocampal cortex and the hippocampus. Interestingly, behavioral and evoked responses to higher-order stimuli were detectable even after responses to first-order stimuli no longer elicited a conditioned response during phase 4 testing. Persisting behavioral and neural responses suggest that while higher-order associations appear to be affected by the strength of first-order associations, they can withstand the concomitant extinction of first-order tones during higher-order training, suggesting at least partial independence of value representations. These findings are consistent with our previous animal studies (Gilboa et al., 2014, 2019) and with the NPRC model that argues for a correspondence between the type of memory representation and the neural substrate that supports it (Hebscher et al., 2019; Gilboa and Moscovitch, 2021). We discuss the implications of these findings to memory representations of value in relation to the animal and human research on higher-order conditioning.

Previously neutral tones acquired value following both first-order and higher-order conditioning, as reflected by pupil dilation and by larger amplitudes of the auditory ERPs. However, the specific temporal window within which significant amplitude increases occurred differed across conditions. Significant CS+/CS– differences occurred later, during early P3, whereas the HO+/HO– difference occurred earlier, during N1. The different auditory components suggest that first-order and higher-order responses can be dissociated by distinct pre-attentive or peri-attentive neural responses because these auditory components are believed to reflect discrete processes. The early P3 component is thought to reflect peri-attentive mechanisms that are responsive to stimulus frequency and intensity whereas the N1 component is thought to reflect pre-attentive sensory elements of auditory selective attention that are responsive to sudden sound changes in the environment (Winkler et al., 2013; Remijn et al., 2014). These characterizations of the P3 and N1 components suggest that responses to CS+ in our study reflect the highly aversive associated intensity of the US+, whereas responses to higher-order stimuli are driven by an early attentional orientation to the auditory features of the response. This early orientation may reflect learning in which stimuli can become represented as contextual stimuli, helping the animal determine in which context a CS:US contingency is active (Honey and Watt, 1999). The earlier response to higher-order than first-order acquired values is reminiscent of findings in smokers (Littel and Franken, 2012) in whom visual ERPs for smoke-related stimuli was later (300 ms) than ones to recently acquired higher-order conditioned stimuli (200–280 ms). Note that while the order is similar, observed ERPs in our study appear much earlier for both first-order and higher-order responses. This may be related to a combination of differences in modality (visual vs. auditory), valance (appetitive vs. aversive), significance (meaningful vs. arbitrary) and strength of first order associations (years of addiction vs. experimental session). Nonetheless, it is noteworthy that in both cases responsivity to higher-order conditioned stimuli appeared earlier than first-order conditioned stimuli. Animal electrophysiology may shed more light on this finding.

First-order and higher-order conditioned responses also differed with respect to the estimated neuroanatomical structures that generated the significant ERP clusters. First-order ERP’s were source estimated to the amygdala, and prefrontal cortex, aligning with human and rodent models which have implicated the amygdala and prefrontal cortex in acquisition and extinction of first-order learning (Lindgren et al., 2003; Holland, 2016; Ebrahimi et al., 2019). Higher-order ERP’s were source estimated the anterior temporal lobe, hippocampus and parahippocampus. These findings align with our previous rodent study that the hippocampus is critical for higher-order but not first-order conditioning (Gilboa et al., 2014). It is consistent with models of memory that posit that the nature of the representation determines the neural substrates engaged during encoding and retrieval (Hebscher et al., 2019; Gilboa and Moscovitch, 2021), contrary to dichotomous memory systems views (e.g. Squire and Zola, 1996). It should be noted that source estimation in our study is limited and thus should be interpreted with caution. Nonetheless, these findings are useful to inform future studies that examine higher-order conditioning using methods with high spatial resolution.

We based our ERP analysis of first-order conditioned associations on phase 3 (higher-order conditioning) because pupil dilation effects for CS+ were still detectable, which were no longer present during phase 4. Interestingly, despite the lack of differentiated pupil responses, CS+/CS– differences in ERPs in phase 4 were still observed in the same ERP component with a broadly similar scalp distribution as in phase 3 (Supplementary Figure 5). The early P3 occurred slightly earlier at 200–255 ms and was source estimated to the right insula and right putamen, consistent with prior research on aversive conditioning (Seymour et al., 2004). While ERPs in phase 3 were measured purely during CS+/CS– tone presentation, the trial as a whole nonetheless entailed other processes as well, such as HO acquisition and possibly CS+ extinction, which may account for these differences.

The current experiment used aversive conditioning, whereas our previous study with rodents used appetitive conditioning with a conflicting aversive contingency. In that previous study, if rodents entered the reward chamber they would receive very mild shock creating incentive to approach only under high certainty (Gilboa et al., 2014). This highlights two important questions that future work would need to examine: what differences we might see when comparing appetitive and aversive higher-order conditioning and what role might conflicting contingencies play in higher-order learning. Previous work in both humans and animal models has shown the importance of the hippocampus for approach-avoidance conflict decision making (Ito and Lee, 2016) consistent with hippocampal involvement in representing complex associations between stimuli (Olsen et al., 2012).

Higher-order associations had been acquired and recalled as shown by a greater pupil response to HO+ compared to HO- in phase 5. We infer that, once acquired, higher-order responses may be at least partially independent of first-order responses to value, because the pupil responses were present in phases 2 and 3 but were no longer observable during phase 4. It appears that despite our efforts to strengthen first-order associations by intensive phase 1 training, (i.e. a night of sleep-enhanced consolidation, phase 2 re-training, and 2 US reminders in phase 4), first order responses had been extinguished during training of higher-order associations, a typical concomitant response to higher-order conditioning (Gewirtz and Davis, 2000). ERP effects during phase 4 suggest that first-order memory engrams were still present but were probably not sufficient to produce detectable pupil responses. Expression of independent first-order and higher-order associations align with previous studies in which independent higher-order associations are expressed after extinction of first-order associations (Pavlov, 1927; Rizley and Rescorla, 1972; Rashotte et al., 1977; Rescorla, 1979; Cole et al., 1995), and in studies where elevated skin conductance responses to higher-order stimuli remained even after extinction of first-order associations (Davey and Arulampalam, 1982). It may be that similar to our findings, residual neural engrams were also present in rodents and humans in these earlier studies, but that it was insufficient to drive overt behavioral responses.

The sequence of stimulus presentation during higher-order conditioning may partially account for the dissociations between higher-order and first-order associations in our experiment. First-order association pairs were arranged in a forward sequence so that the CS precedes the US, whereas the higher-order association pairs were arranged in a backward sequence so that the HO follows the, now value carrying, CS. In other words, the HO was presented when participants expected the US to appear and this may have contributed to the persistence of higher-order associations. While several human higher-order conditioning studies use classical Pavlovian training procedures in which HO predicts a previously conditioned CS (see Lee, 2021 for review), different conditioning procedures have been used including sequential conditioning where distal (HO) and proximal (CS) both precede the US (Seymour et al., 2004; Pauli et al., 2019) and also different combinations of backward conditioning as in our study (Prével et al., 2016, 2019). Higher-order learning in our paradigm was modeled after an animal higher-order conditioning study that used the same presentation structure (Gilboa et al., 2014). That study also found a dissociation between first-order and higher-order behavioral responses as well as the neuroanatomical structures involved in both processes. Other rodent work has used backwards higher-order conditioning with a forwards first-order trace conditioning and found a dissociation between higher-order and first-order associations (Cole et al., 1995) although the authors suspected predictable stimulus durations provided temporal information that may have confounded CS2 responses. We varied CS duration, as recommended by Cole et al. (1995), avoiding this potential confound. In line with our study, previous human higher-order conditioning studies have used backwards sequence higher-order associations and showed that expression of higher-order CRs was maintained after first-order stimuli no longer elicit the CR (Prével et al., 2019). Note however, that in the Prével et al. (2019) study, first order associations were also trained with backward conditioning. These authors suggest that backwards conditioning may lead to an associative structure that is resistant to extinction of first-order associations and that individuals may learn to associate stimuli to form flexible representations of their environment as participants’ responses appear to index bidirectional relationships rather than linear chains (Honey and Watt, 1999; Arcediano et al., 2005; Molet et al., 2010; Prével et al., 2016, 2019) consistent with representational characteristics of hippocampal memory traces (Gilboa and Moscovitch, 2021).

Results from this study should be interpreted with caution and may not be generalizable due to the small sample size of participants that were included. Further studies should be conducted to examine this phenomenon. Moreover, we cannot completely rule out that some participants may have developed awareness of the associative nature of the experiment. While this is unlikely given the early nature of the ERP differences and lack of change in explicit ratings of tone valence, further studies should probe the issue of awareness more extensively.

## Conclusion

In conclusion, this experiment demonstrates that memory for first-order and higher-order conditioned associations reflect distinct pre-attentive and peri-attentive electrophysiological responses. These likely originate from distinct sources. The findings are consistent with literature implicating the amygdala and prefrontal cortex in first-order conditioning and extinction. They are also in line with recent animal studies that have implicated the hippocampus, specifically in higher-order conditioning and suggest its involvement may be rapid.

## Data Availability Statement

The raw data supporting the conclusions of this article will be made available by the authors, without undue reservation.

## Ethics Statement

The studies involving human participants were reviewed and approved by Rotman Research Institute/Baycrest Hospital Research Ethics Board. The patients/participants provided their written informed consent to participate in this study. Written informed consent was obtained from the individual(s) for the publication of any potentially identifiable images or data included in this article.

## Author Contributions

AG designed the experiment. AW collected the data and conducted the cleaning and basic analysis of EEG data. PD conducted the data cleaning, pre-processing, analysis of pupillometry, and EEG data. AG and PD conducted the analysis and wrote the manuscript. All authors contributed to the article and approved the submitted version.

## Conflict of Interest

The authors declare that the research was conducted in the absence of any commercial or financial relationships that could be construed as a potential conflict of interest.

## Publisher’s Note

All claims expressed in this article are solely those of the authors and do not necessarily represent those of their affiliated organizations, or those of the publisher, the editors and the reviewers. Any product that may be evaluated in this article, or claim that may be made by its manufacturer, is not guaranteed or endorsed by the publisher.

## Footnotes

1 We had planned to test neurological patients for this study before the COVID-19 pandemic, and we would not have enough patients for counterbalancing, and therefore, we did not counterbalance stimuli in this study.

## References

[B1] Aiken S. J. Picton T. W. (2008). Envelope and spectral frequency-following responses to vowel sounds. *Hear. Res.* 245 35–47. 10.1016/j.heares.2008.08.004 18765275

[B2] Arcediano F. Escobar M. Miller R. R. (2005). Bidirectional associations in humans and rats. *J. Exp. Psychol. Anim. Behav. Process.* 31:301. 10.1037/0097-7403.31.3.301 16045385

[B3] Campbell T. Kerlin J. R. Bishop C. W. Miller L. M. (2012). Methods to eliminate stimulus transduction artifact from insert earphones during electroencephalography. *Ear. Hear.* 33:144. 10.1097/AUD.0b013e3182280353 21760513 PMC3214253

[B4] Cole R. P. Barnet R. C. Miller R. R. (1995). Temporal encoding in trace conditioning. *Anim. Learn. Behav.* 23 144–153. 10.3758/bf03199929

[B5] Davey G. C. L. Arulampalam T. (1982). Second-order ‘fear’ conditioning in humans: persistence of CR2 following extinction of CR1. *Behav. Res. Ther.* 20 391–396. 10.1016/0005-7967(82)90099-7 7126121

[B6] Ebrahimi C. Koch S. P. Pietrock C. Fydrich T. Heinz A. Schlagenhauf F. (2019). Opposing roles for amygdala and vmPFC in the return of appetitive conditioned responses in humans. *Transl. Psychiatry* 9 1–12. 10.1038/s41398-019-0482-x 31113931 PMC6529434

[B7] Finke J. B. Roesmann K. Stalder T. Klucken T. (2021). Pupil dilation as an index of Pavlovian conditioning. A systematic review and meta-analysis. *Neurosci. Biobehav. Rev.* 130 351–368. 10.1016/j.neubiorev.2021.09.005 34499928

[B8] Gewirtz J. C. Davis M. (1997). Second-order fear conditioning prevented by blocking NMDA receptors in amygdala. *Nature* 388, 471–474. 10.1038/41325 9242405

[B9] Gewirtz J. C. Davis M. (2000). Using Pavlovian higher-order conditioning paradigms to investigate the neural substrates of emotional learning and memory. *Learn. Mem.* 7 257–266. 10.1101/lm.35200 11040256

[B10] Gilboa A. Moscovitch M. (2021). No consolidation without representation: correspondence between neural and psychological representations in recent and remote memory. *Neuron* 109, 2239–2255. 10.1016/j.neuron.2021.04.025 34015252

[B11] Gilboa A. Sekeres M. Moscovitch M. Winocur G. (2014). Higher-order conditioning is impaired by hippocampal lesions. *Curr. Biol.* 24 2202–2207. 10.1016/j.cub.2014.07.078 25201688

[B12] Gilboa A. Sekeres M. Moscovitch M. Winocur G. (2019). The hippocampus is critical for value-based decisions guided by dissociative inference. *Hippocampus* 29 655–668. 10.1002/hipo.23050 30417959

[B13] Hebscher M. Wing E. Ryan J. Gilboa A. (2019). Rapid cortical plasticity supports long-term memory formation. *Trends Cogn. Sci.* 23, 989–1002. 10.1016/j.tics.2019.09.009 31703929

[B14] Hershman R. Henik A. Cohen N. (2018). A novel blink detection method based on pupillometry noise. *Behav. Res. Methods* 50 107–114. 10.3758/s13428-017-1008-1 29340968

[B15] Hershman R. Henik A. Cohen N. (2019). CHAP: open-source software for processing and analyzing pupillometry data. *Behav. Res. Methods* 51 1059–1074. 30710333 10.3758/s13428-018-01190-1

[B16] Hershman R. Levin Y. Tzelgov J. Henik A. (2021). Neutral stimuli and pupillometric task conflict. *Psychol. Res.* 85 1084–1092. 10.1007/s00426-020-01311-6 32170401

[B17] Holland P. C. (2016). Enhancing second-order conditioning with lesions of the basolateral amygdala. *Behav. Neurosci.* 130:1. 10.1037/bne0000129 26795578 PMC4792661

[B18] Honey R. C. Dwyer D. M. (2021). Higher-order conditioning: what is learnt and how it is expressed. *Front. Behav. Neurosci.* 15:726218. 10.3389/fnbeh.2021.726218 34566595 PMC8462663

[B19] Honey R. C. Watt A. (1999). Acquired relational equivalence between contexts and features. *J. Exp. Psychol. Anim. Behav. Process.* 25:324. 10.1037/0097-7403.25.3.324 9679308

[B20] Ito R. Lee A. C. (2016). The role of the hippocampus in approach-avoidance conflict decision-making: evidence from rodent and human studies. *Behav. Brain Res.* 313 345–357. 10.1016/j.bbr.2016.07.039 27457133

[B21] Jeffreys H. (1961). *The Theory of Probability.* Oxford: Oxford University Press.

[B22] Kamil A. C. (1969). Some parameters of the second-order conditioning of fear in rats. *J. Compar. Physiol. Psychol.* 67:364. 10.1037/h0026782 5787387

[B23] Korn C. W. Staib M. Tzovara A. Castegnetti G. Bach D. R. (2017). A pupil size response model to assess fear learning. *Psychophysiology* 54 330–343. 10.1111/psyp.12801 27925650 PMC5324687

[B24] Lee J. C. (2021). Second-order conditioning in humans. *Front. Behav. Neurosci.* 15:672628. 10.3389/fnbeh.2021.672628 34305546 PMC8295922

[B25] Lindgren J. L. Gallagher M. Holland P. C. (2003). Lesions of basolateral amygdala impair extinction of CS motivational value, but not of explicit conditioned responses, in pavlovian appetitive second-order conditioning. *Euro. J. Neurosci.* 17 160–166. 10.1046/j.1460-9568.2003.02421.x 12534980

[B26] Littel M. Franken I. H. (2012). Electrophysiological correlates of associative learning in smokers: a higher-order conditioning experiment. *BMC Neurosci.* 13:8. 10.1186/1471-2202-13-8 22235938 PMC3277456

[B27] Luettgau L. Porcu E. Tempelmann C. Jocham G. (2021). Reinstatement of cortical outcome representations during higher-order learning. *Cereb. Cortex* 32 93–109. 10.1093/cercor/bhab196 34383017

[B28] Molet M. Jozefowiez J. Miller R. R. (2010). Integration of spatial relationships and temporal relationships in humans. *Learn. Behav.* 38 27–34. 10.3758/LB.38.1.27 20065346 PMC2846430

[B29] Molholm S. Martinez A. Ritter W. Javitt D. C. Foxe J. J. (2005). The neural circuitry of pre-attentive auditory change-detection: an fMRI study of pitch and duration mismatch negativity generators. *Cereb. Cortex* 15 545–551. 10.1093/cercor/bhh155 15342438

[B30] Ojala K. E. Bach D. R. (2020). Measuring learning in human classical threat conditioning: translational, cognitive and methodological considerations. *Neurosci. Biobehav. Rev.* 114 96–112. 10.1016/j.neubiorev.2020.04.019 32343982

[B31] Olsen R. K. Moses S. N. Riggs L. Ryan J. D. (2012). The hippocampus supports multiple cognitive processes through relational binding and comparison. *Front. Hum. Neurosci.* 6:146. 10.3389/fnhum.2012.00146 22661938 PMC3363343

[B32] Papesh M. H. Pinto J. D. G. (2019). Spotting rare items makes the brain “blink” harder: evidence from pupillometry. *Attent. Percept. Psychophys.* 81 2635–2647. 10.3758/s13414-019-01777-6 31222658 PMC6858538

[B33] Pauli W. M. Gentile G. Collette S. Tyszka J. M. O’Doherty J. P. (2019). Evidence for model-based encoding of Pavlovian contingencies in the human brain. *Nat. Commun.* 10 1–11. 10.1038/s41467-019-08922-7 30846685 PMC6405831

[B34] Pavlov I. P. (1927). *Conditioned Reflexes: an Investigation of the Physio-logical Activity of the Cerebral Cortex.* New York: Dover Publications.

[B35] Prével A. Rivière V. Darcheville J. C. Urcelay G. P. (2016). Conditioned reinforcement and backward association. *Learn. Motivat.* 56 38–47. 10.1016/j.lmot.2016.09.004

[B36] Prével A. Rivière V. Darcheville J. C. Urcelay G. P. Miller R. R. (2019). Excitatory second-order conditioning using a backward first-order conditioned stimulus: A challenge for prediction error reduction. *Q. J. Exp. Psychol.* 72 1453–1465. 10.1177/1747021818793376 30041571

[B37] R Core Team (2014). *R: a Language and Environment for Statistical Computing.* Vienna: R Foundation for Statistical Computing.

[B38] Rashotte M. E. Griffin R. W. Sisk C. L. (1977). Second-order conditioning of the pigeon’s keypeck. *Anim. Learn. Behav.* 5 25–38.

[B39] Reinhard G. Lachnit H. (2002). Differential conditioning of anticipatory pupillary dilation responses in humans. *Biol. Psychol.* 60 51–68. 10.1016/s0301-0511(02)00011-x 12100845

[B40] Remijn G. B. Hasuo E. Fujihira H. Morimoto S. (2014). An introduction to the measurement of auditory event-related potentials (ERPs). *Acoust. Sci. Technol.* 35 229–242. 10.1250/ast.35.229

[B41] Rescorla R. A. (1979). Aspects of the reinforcer learned in second-order Pavlovian conditioning. *J. Exp. Psychol.* 5 79–95. 10.1037/0097-7403.5.1.79 528880

[B42] Rescorla R. A. (1982). Simultaneous second-order conditioning produces SS learning in conditioned suppression. *J. Exp. Psychol. Anim. Behav. Process.* 8:23. 10.1037/0097-7403.8.1.23 7057142

[B43] Rizley R. C. Rescorla R. A. (1972). Associations in second-order conditioning and sensory preconditioning. *J. Compar. Physiol. Psychol.* 81 1–11. 10.1037/h0033333 4672573

[B44] Rouder J. N. Morey R. D. Speckman P. L. Province J. M. (2012). Default Bayes factors for ANOVA designs. *J. Mathemat. Psychol.* 56 356–374.

[B45] Seymour B. O’Doherty J. P. Dayan P. Koltzenburg M. Jones A. K. Dolan R. J. (2004). Temporal difference models describe higher-order learning in humans. *Nature* 429 664–667. 10.1038/nature02581 15190354

[B46] Squire L. R. Zola S. M. (1996). Structure and function of declarative and nondeclarative memory systems. *Proc. Natl. Acad. Sci.* 93 13515–13522. 10.1073/pnas.93.24.13515 8942965 PMC33639

[B47] The Jamovi Project (2020). *Jamovi (Version 1.2) [Computer Software].* Available online at: https://www.jamovi.org (accessed December 12, 2021).

[B48] Wimmer G. E. Shohamy D. (2012). Preference by association: how memory mechanisms in the hippocampus bias decisions. *Science* 338, 270–273. 10.1126/science.1223252 23066083

[B49] Winkler I. Denham S. Escera C. (2013). “Auditory Event-related Potentials” in *Encyclopedia of Computational Neuroscience.* eds Jaeger D. Jung R. (New York: Springer). 10.1007/978-1-4614-7320-6_99-1

[B50] Woodman G. F. (2010). A brief introduction to the use of event-related potentials in studies of perception and attention. *Attent. Percept. Psychophys.* 72 2031–2046. 10.3758/APP.72.8.2031 21097848 PMC3816929

