# Supplementary material for: Early Auditory Event Related Potentials Distinguish Higher-Order From First-Order Aversive Conditioning
Source: Front Behav Neurosci. 2022 Feb 11;16:751274. doi: 10.3389/fnbeh.2022.751274 (PMC8879319; doi:10.3389/fnbeh.2022.751274)
Supplement: Supplementary file 1 [file Data_Sheet_1.pdf]

Supplementary Material

Supplementary Analysis 1 – Tone Rating Task

Participants rated aversiveness of stimuli prior to and after the Phase 1: First-Order Conditioning, Phase 2: First Order Reminder and Phase 3: Higher-Order Conditioning tasks. Analyses were conducted using 3 three-way repeated measures ANOVAs. Estimated marginal means and standard errors are reported below.

Phase 1: First Order Conditioning. A three-way repeated measures ANOVA was conducted to compare the effect of the stimuli type (CS or US), value (+: aversive/ -: neutral) on subjective tone ratings pre and post the FOC task.

There was a significant two-way interaction between valence and order, $F(1,10) = 37.89$, $p < .001$. A follow-up analyses revealed that the US+ tone (*EMM* = 8.50 , *SEM* = .47) was rated significantly more aversive than all other stimuli, $p_{bonferroni} < .001$. No other tones were rated significantly different from each other, all .

There was no main effect of time as pre- and post- task subjective tone ratings did not differ, *all p's* > .05.

Phase 2: First Order Reminder. A three-way repeated measures ANOVA was conducted to compare the effect of the stimuli type (CS or US), value (+: aversive/ -: neutral) on subjective tone ratings pre and post the FOC task.

There was a significant two-way interaction between valence and order, $F(1,10) = 14.09$, $p = .004$. A follow-up analyses revealed that the US+ tone (*EMM* = 8.50, *SEM* = .47), was rated significantly more aversive than all other stimuli, $p_{bonferroni} < .001$. No other tones were rated significantly different from each other, all $p_{bonferroni}$'s > .05 .

There was no main effect of time as pre- and post- task subjective tone ratings did not differ, $p > .05$.

Phase 3: Higher Order Conditioning. A three-way repeated measures ANOVA was conducted to compare the effect of the order (CS or HO), value (+/-) on subjective tone ratings pre and post the Higher-Order Conditioning. There was a significant two-way interaction between valence and order, $F(1,13) = 9.46$, $p = .009$. A follow-up analyses revealed that, the CS- tone (*EMM* = 2.21, *SEM* = .48), was rated as significantly more aversive than HO- tones, (*EMM* = 3.96 , *SEM* = .48), $p = .004$.

There was no main effect of time as pre- and post- task subjective tone ratings did not differ, $p > .05$.

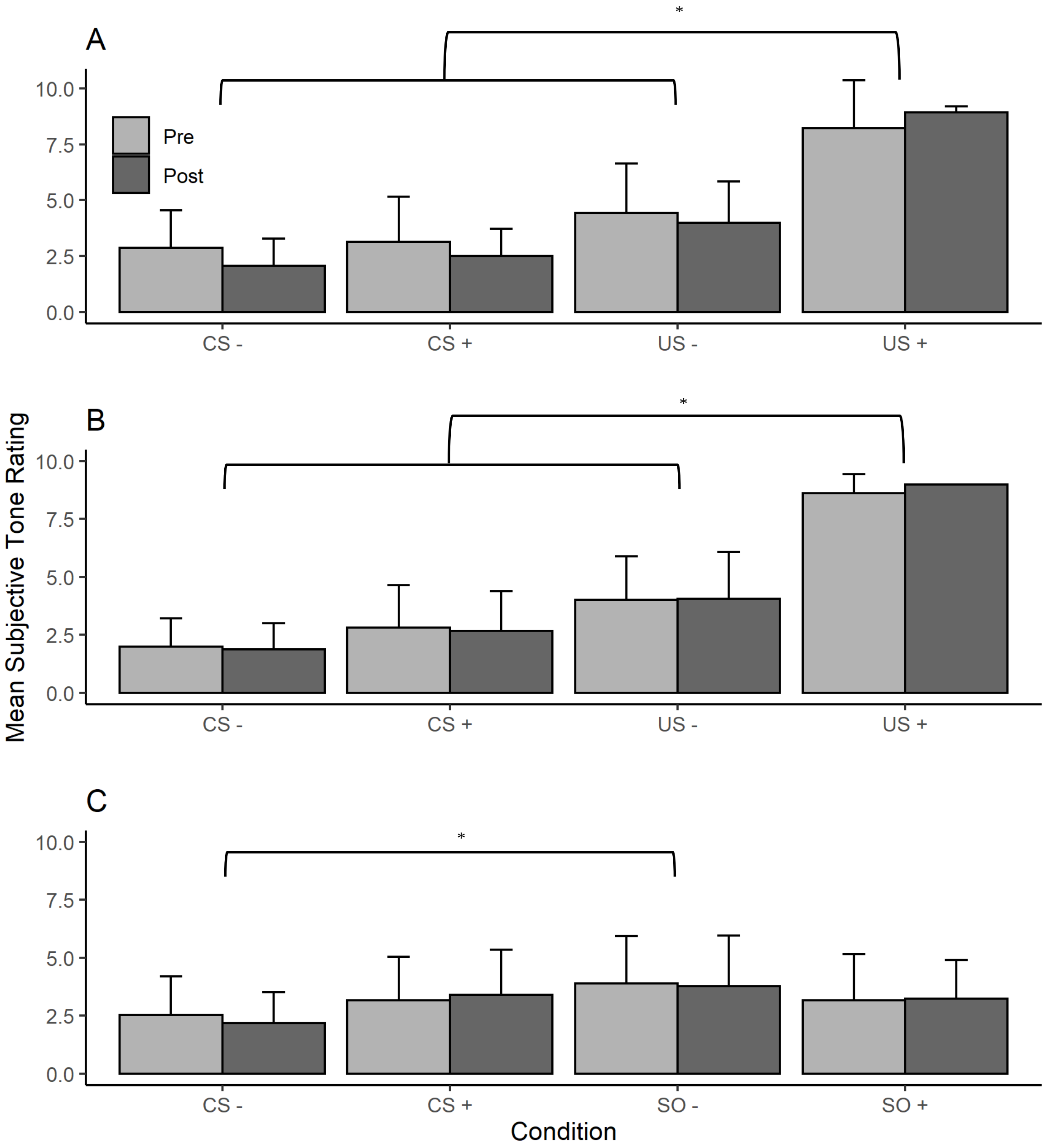

Supplementary Figure 1. Comparison of tone ratings of stimuli pre and post conditioning using three mixed measures 2 x 2 ANOVA. No significant within-subjects main effects (**A.** Phase 1: First-Order Conditioning. **B.** Phase 2: First-Order Reminder **C.** Phase 3: Higher-Order Conditioning. US: unconditioned stimulus, CS: conditioned stimulus, + : aversive value/paired with aversive value stimulus, - : neutral value/paired with neutral value stimulus.

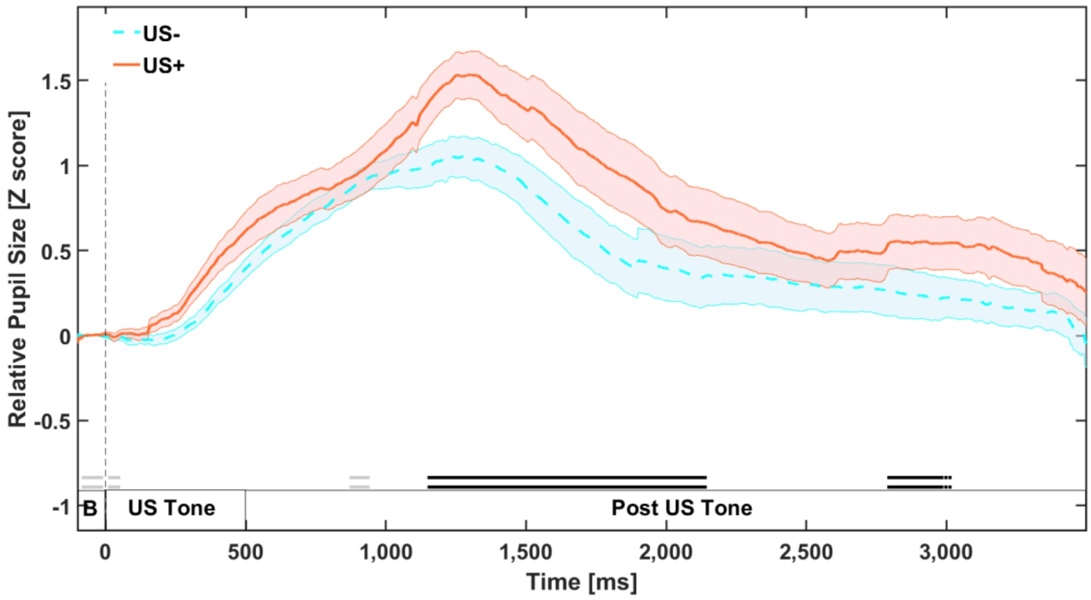

Supplementary Figure 2. Mean relative pupil dilation (z-score) responses during the first 7 presentations of US+ and US-. Meaningful differences (BF10 ≥ 3) are indicated by an opaque double line near the bottom of the figure, above the segment labels. Evidence of meaningful difference (BF10 ≥ 3) can be observed from approximately 1200 ms – 2200 ms and from 2750 ms – 3000 ms. Evidence in favor of the null hypothesis is indicated by faded double line near the bottom of the figure, which can be observed from 0 ms – 100 ms and 800 ms – 900 ms. The dark lines in colour represents the mean of that condition (orange solid line for US+ trials and blue dashed line for US- trials). The lighter colour bands surrounding the darker coloured lines represent Standard Error of the Mean. The baseline is 100 ms and represented as B.

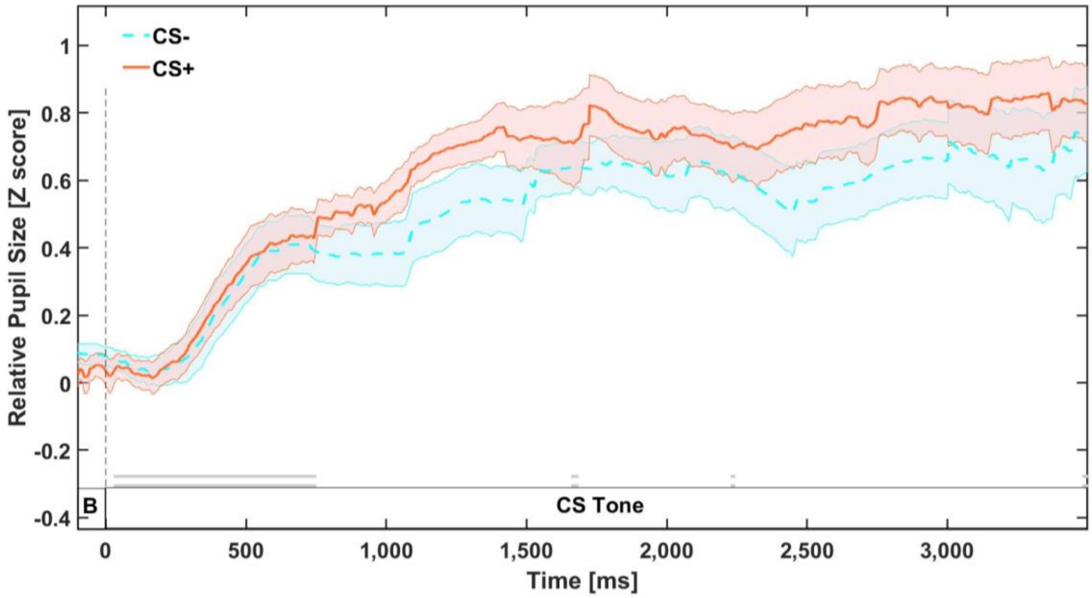

Supplementary Figure 3. Mean relative pupil dilation (z-score) responses during the first 7 presentations of CS+ and CS-. Evidence in favor of the null hypothesis is indicated by light grey double line near the bottom of the figure (BF01 ≥ 3) and can be seen from approximately 50 ms – 750 ms. The lines in colour represents the mean of that condition (orange solid line for CS+ trials and blue dashed line for CS- trials). The lighter colour bands surrounding the darker coloured lines represent Standard Error of the Mean. The baseline is 100 ms and represented as B.

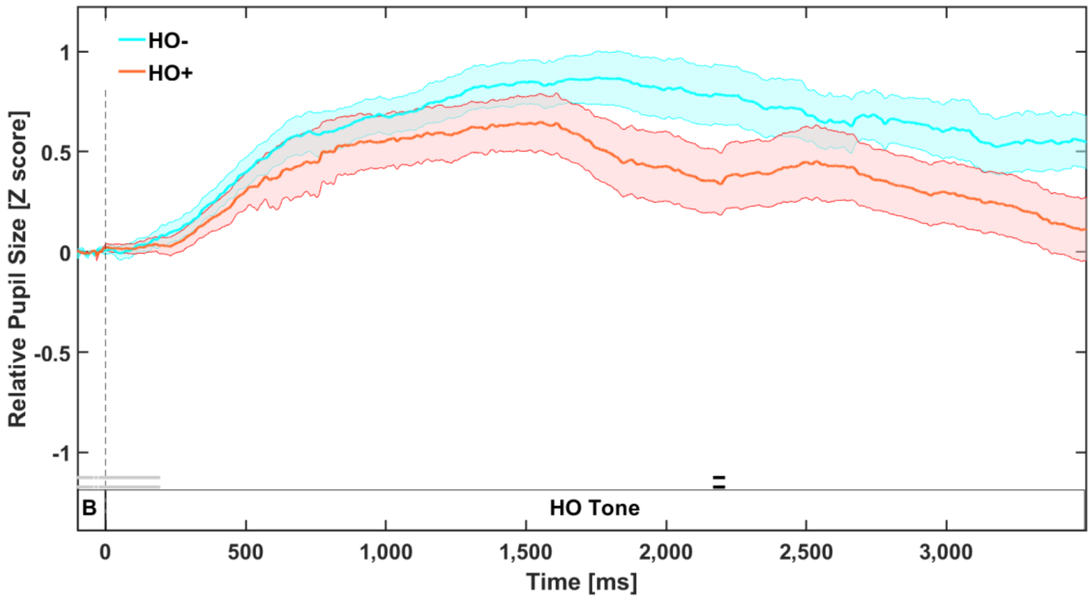

Supplementary Figure 4. Mean relative pupil dilation (z-score) responses during the first 7 presentations of HO+ and HO-. Meaningful differences (BF10 ≥ 3) are indicated by an opaque double line near the bottom of the figure, above the segment labels. We can see that HO- tones elicited a brief meaningfully greater pupil dilation from HO+ at 2200 ms – 2215 ms, i.e. the reverse direction as what was found after training. Evidence in favor of the null hypothesis (BF01 ≥ 3) is indicated by faded double line near the bottom of the figure, which can be observed from approximately 0 ms – 250 ms. The dark line in colour represents the mean of that group (solid orange line for HO+ trials and dashed blue line for HO- trials). The lighter colour bands surrounding the darker coloured lines represent Standard Error of the Mean. The baseline is 100 ms and represented as B.

Supplementary Analysis 2 – Neural Responses to First-Order Stimuli Presented during Phase 4: ERP

While there was little evidence of first-order stimuli eliciting pupil dilation conditioned responses during Phase 4, we examined the neural responses to determine if the stimuli had been fully or partially extinguished. Interestingly, the patterns of neural responses observed are similar to the ones observed for first-order stimuli presented during phase 3.

Permutation based analyses correcting for temporal and spatial extents of the ERP waveforms revealed a significant difference from 200 ms to 255 ms, with a greater positive peak in response to CS+ tones. This cluster encompassed electrodes bilaterally in frontal (F1, Fz, F2, F4, FC1, FCz, FC2, FC4, FC3, FC1), central (C3, C1, Cz, C2, C4) and parietal areas (CP3, CP1, CPz, CP2, P3, P1, Pz). Within the temporal extension of the significant cluster, the peak response was centered on frontal, and central electrodes (FCz, Cz) throughout the response, cluster-based statistics, $p<.0001$.

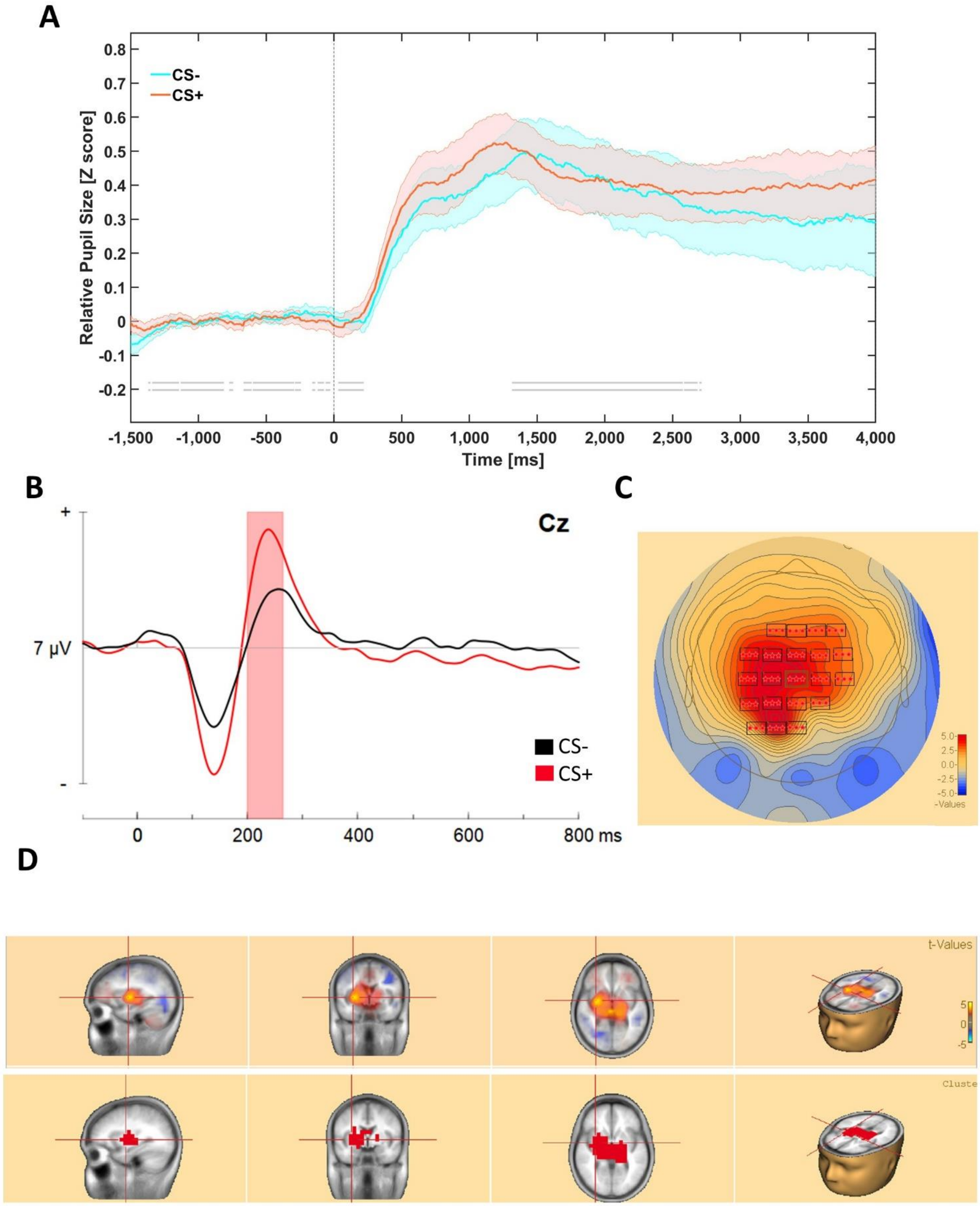
A
CS-
CS+
Relative Pupil Size [Z score]
0.8
0.7
0.6
0.5
0.4
0.3
0.2
0.1
0
-0.1
-0.2
-1,500
-1,000
-500
0
500
1,000
1,500
2,000
2,500
3,000
3,500
4,000
Time [ms]
B
Cz
+
7 µV
-
CS-
CS+
0
200
400
600
800 ms
C
5.0
2.5
0.0
-2.5
-5.0
-Values
D
t-Values
5
0
-5
Cluste

Supplementary Figure 5. **A.** Mean relative pupil dilation (z-score) responses during first order tones in phase 4, between -1500 ms to 4000 ms. Meaningful differences ($BF10 \geq 3$) would be indicated by an opaque double line near the bottom of the figure, above the segment labels, however no evidence to suggest meaningful differences was observed during this phase. Evidence in favor of the null hypothesis is indicated by faded double line near the bottom of the figure. The dark line in colour represents the mean of that condition (solid orange line for CS+ trials and dashed blue line for CS- trials). The lighter colour bands surrounding the darker coloured lines represent Standard Error of the Mean. **B.** ERP response to first-order stimuli presented during phase 4: first-order test at Cz. Bootstrap cluster analyses revealed a significant difference from 200ms to 255ms. **C.** Mean potential distribution maps average across participants (n=15) at scalp level. The significant positive modulation peaks bilaterally in frontal-parietal areas. D. Source estimation analysis was conducted from 200ms to 255 ms encompassing the significant greater modulation identified in source estimation. A positive significant source was identified right insula and encroached onto the right putamen.

Supplementary Analysis 3 – Additional Pupillometry Analyses

We calculated the 95% credible interval for mean paired difference as well as a paired-samples t-test for the standard error of the mean (S.E.M.) of the paired difference, standard deviation of the mean paired difference and the probability of the difference being different than 0 (Table 1).

Table 1. Probability of the estimate for the mean differences being different than zero, S.E.M. of the paired difference, standard deviation of the mean paired difference and the 95% credible confidence interval for mean paired difference.

Phase	n	Probability of estimate for the mean differences (CS+ - CS-) being different than 0	S.E.M. of the mean paired difference	Standard Deviation of the mean paired difference	95% Credible Interval for mean paired difference
1	11	.023*	0.06	0.21	[0.04; 1.36]
2	13	< .001*	0.06	0.21	[0.36; 1.81]
3	12	.006*	0.07	0.25	[0.19; 1.56]
4	12	.240	0.05	0.18	[-0.23; 0.85]
5	11	.011*	0.06	0.19	[0.12; 1.51]

* denotes significant at .05

JASP Team (2021). JASP (Version 0.16) [Computer software].
